# High-definition transcranial direct current stimulation of the right inferior parietal modulates oscillatory activity in higher-order regions serving attention reorientation

**DOI:** 10.1162/IMAG.a.1054

**Published:** 2025-12-09

**Authors:** Tara D. Erker, Yasra Arif, Jason A. John, Kellen M. McDonald, Hannah J. Okelberry, Kennedy A. Kress, Giorgia Picci, Tony W. Wilson

**Affiliations:** Institute for Human Neuroscience, Boys Town National Research Hospital, Boys Town, NE, United States; Department of Pharmacology & Neuroscience, Creighton University, Omaha, NE, United States

**Keywords:** magnetoencephalography (MEG), Posner cueing task, attention, neural oscillations

## Abstract

Attentional reorientation is crucial to navigating the world and relies heavily on the parietal cortex. Several studies have shown that transcranial direct current stimulation (tDCS) has an effect on attentional reorientation; however, these studies mainly focused on the difference between anodal and cathodal stimulation. In this study, we investigated the role of left versus right inferior parietal cortices in attention reorientation by applying high-definition tDCS (HD-tDCS) to these regions in 39 healthy adults (age 19–33 years) for 20 minutes in 3 separate sessions (left active, right active, and sham). Following stimulation, participants completed a modified Posner task during magnetoencephalography (MEG). Significant neural responses at the sensor level across all conditions were then imaged using a beamformer, and the whole-brain, voxel-wise maps were probed for task condition and stimulation montage effects. Our neural findings showed significant stimulation montage by task condition interactions that were multispectral and involved the left frontal eye fields, right inferior parietal cortex, and left anterior prefrontal cortex. We also observed main effects of stimulation montage in the theta and beta ranges, main effects of task condition that were consistent with previous studies of attentional reorienting, and a significant neurobehavioral relationship between theta oscillations in the left frontal eye fields and accuracy. Overall, our findings indicate that HD-tDCS of the inferior parietal cortices modulates several brain regions that are important for attentional reorientation.

## 1 Introduction

The ability to redirect attention toward relevant stimuli is crucial to many daily activities, including driving, sports, and even basic cognitive tasks. Attentional reorientation has been studied widely in the past using the Posner task, which involves a cue appearing slightly before a target stimulus to bias the participant’s attention toward or away from the upcoming target. The cue can either be on the same side as the target (i.e., a valid trial) or on the opposite side (i.e., an invalid trial; Posner, 1980). Invalid trials force participants to reorient their attention from the biased side toward the target, which makes it an ideal task for capturing the brain regions activated during attentional reorientation. Broadly, frontoparietal regions are known to be essential for attentional control, and have been split into two spatially distinct but functionally interacting networks, the dorsal attention network (DAN) and the ventral attention network (VAN; Arif, Spooner, Wiesman, Embury, et al., 2020; Corbetta et al., 2008; Proskovec et al., 2018; Roy et al., 2015; Spadone et al., 2021; Vossel et al., 2014). The DAN is considered to be primarily responsible for the top–down processing of sensory information that is pertinent to current goals (Corbetta et al., 2008; Proskovec et al., 2018; Roy et al., 2015; Spadone et al., 2021), and comprises the bilateral superior parietal lobes, the intraparietal sulcus, and the frontal eye fields (Corbetta et al., 2008; Proskovec et al., 2018; Roy et al., 2015). The DAN often becomes active before the onset of a stimulus due to the expectation of stimulus presentation in a particular location, such as during the cue period of the Posner task (Corbetta et al., 2008; Proskovec et al., 2018). The VAN, made up of the temporoparietal junction (TPJ) and the ventral frontal cortex, is most commonly activated in conjunction with the DAN when behaviorally relevant stimuli appear outside the current focus of attention, as in invalid trials of the Posner task (Corbetta et al., 2008; Proskovec et al., 2018; Roy et al., 2015; Spadone et al., 2021). These two networks have been shown to be highly related in resting-state and task-evoked activation, mainly through connections in the middle frontal gyrus and/or TPJ (Corbetta et al., 2008; Vossel et al., 2014). The communication and connectivity between the DAN and the VAN allow for flexible recruitment of each network so attention can be reoriented swiftly and accurately.

As mentioned above, frontoparietal regions are heavily involved in attentional reorientation, with the parietal lobe being particularly important to attention as it is included in the DAN. The parietal cortex has also been shown to be active during switching tasks and is known to be critical for stimulus–response linking (Corbetta & Shulman, 2002; Proskovec et al., 2019). Several studies have shown that neural activity in the parietal lobe increases following invalid cues, suggesting that this region is heavily active in attention reorientation (Arif, Spooner, Wiesman, Embury, et al., 2020; Proskovec et al., 2018; Vossel et al., 2006). Given the importance of frontoparietal regions to attention function, past studies have probed the effects of using noninvasive stimulation, such as repetitive transcranial magnetic stimulation (rTMS) or transcranial direct-current stimulation (tDCS), to target these regions. For example, several studies have shown that rTMS of the parietal cortex affects attentional task performance (Capotosto et al., 2012; Middag‐van Spanje et al., 2022; Rushworth & Taylor, 2006; Yeager et al., 2021), with inhibitory rTMS of the right parietal cortex leading to slower reaction times and lower accuracy relative to stimulation of the left parietal cortex (Rushworth & Taylor, 2006; Yeager et al., 2021). Along with worsened performance, Capotosto et al. (2012) found a decrease in the neural P3 response to invalid targets following right parietal excitatory rTMS, which they posited was due to the stimulation interfering with the normal alpha oscillations observed in the parietal cortex.

In contrast to rTMS, tDCS alters the local ionic environment in the brain without inducing action potentials to change the response threshold or excitability of local neural populations (Liebetanz, 2002; M. A. Nitsche et al., 2003; M. Nitsche & Paulus, 2001). As such, tDCS has been used to study the parietal lobe in many previous studies (Da Costa Leal et al., 2022; Datta et al., 2009; Edwards et al., 2013; Erker et al., 2024; Lo et al., 2019; Minamoto et al., 2014). While several of these studies have examined the effects of parietal tDCS on attention, the researchers utilized saline-soaked sponge electrodes, which are known to cause more widespread modulation (Datta et al., 2009; Edwards et al., 2013). A new approach known as high-definition (HD-tDCS) or multielectrode tDCS uses an array of stimulating electrodes to better focus the modulation to the intended target, and limit stimulation of the surrounding regions, which can sometimes dampen or even reverse the effect (Datta et al., 2009; Edwards et al., 2013). Thus, utilizing HD-tDCS to selectively stimulate the parietal lobe, which is known to be a critical region for attentional function, could provide key information about the importance of right versus left parietal cortices to attentional reorientation in healthy adults.

In this study, we examined whether applying HD-tDCS to the left or right inferior parietal cortices more strongly affected neural activity and task performance during a Posner task. Specifically, we utilized a three visit, double-blinded, crossover design where each participant received 20 minutes of HD-tDCS to the left or right parietal, or sham stimulation, with each stimulation session being followed by a high-density magnetoencephalography (MEG) recording during which the participant performed a modified version of the Posner attention reorientation task. The order of stimulation conditions was pseudorandomized across the sample. Based on the stronger right lateralization of attention networks, we hypothesized that active stimulation of the right inferior parietal cortices would be associated with stronger neural oscillations during task performance relative to left parietal stimulation. Further, we expected these neural effects to be expressed across task conditions in some brain regions and to vary by task condition (e.g., stronger during invalid trials) in other cortical regions, especially those commonly associated with the DAN.

## 2 Methods

### 2.1 Participants

We enrolled 39 right-handed healthy adults (21 females) with a mean age of 25.6 years (range: 19–33 years) in the study. Exclusionary criteria included any medical illness affecting CNS function (e.g., HIV/AIDS), any neurological or psychiatric disorder, history of head trauma, current substance use, and the standard exclusion criteria for MEG and MRI data acquisition (e.g., no ferromagnetic implants). All experimental procedures conformed to the standards set by the *Declaration of Helsinki*. The study protocol was approved by the Boys Town National Research Hospital’s Institutional Review Board (IRB). A full description of the study was given to all participants, followed by written informed consent, which adhered to the guidelines set forth by the IRB.

### 2.2 High-definition transcranial electrical stimulation

**Fig. 1.**
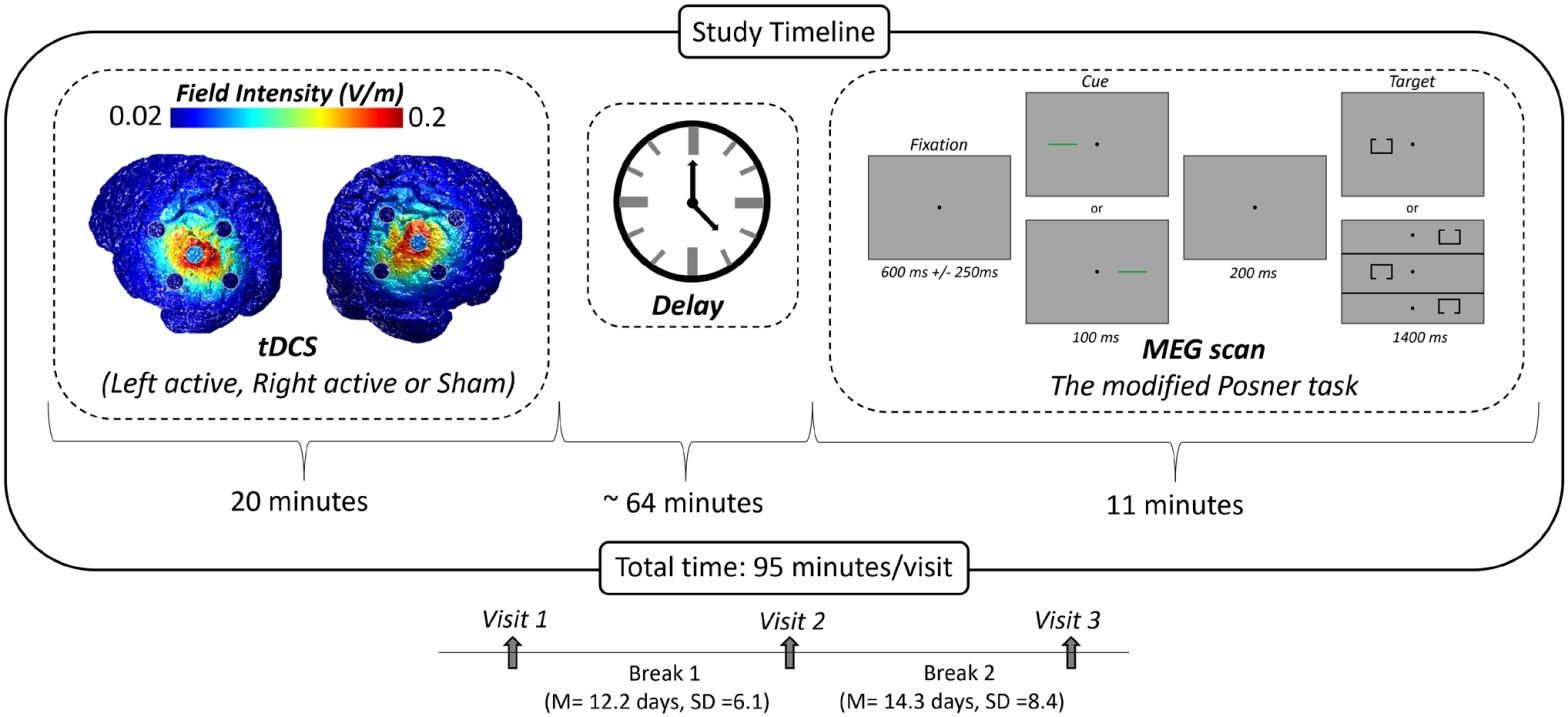
Visit timeline, current flow model, and experimental paradigm. Participants received 20 minutes of HD-tDCS over the left or right inferior parietal cortices across three study visits. Stimulation montages (i.e., anodal left, anodal right, and left/right sham) were pseudorandomized across the three visits, each separated by at least a week and occurring at the same time of day. Current distribution modeling revealed focal stimulation of the left and right inferior parietal cortices (left). Following HD-tDCS, participants completed an attention reorientation paradigm during MEG recording (right). The total visit time from the beginning of stimulation to the end of the MEG task was approximately 95 minutes.

An HD-tDCS 4 × 1 electrode configuration with a central anode surrounded by four cathodes (Soterix Medical, New York) was applied to the left and right inferior parietal cortices using the international 10/20 system (Jasper, 1958; Klem et al., 1999). Based on the Okamoto et al. transformations of the scalp-based international 10/20 system into MNI space, the central anode was placed on P3 (with the surrounding cathodes at P1, P5, PO3, CP3) and P4 (with the surrounding cathodes at P2, P6, PO4, CP4), corresponding to the left and right inferior parietal cortices, respectively (Okamoto et al., 2004; Okamoto & Dan, 2005). To estimate the focality and intensity of these HD-tDCS configurations, current density modeling was conducted with the ROAST software. This modeling utilized a standardized template brain and showed peak stimulation within the targeted areas of the parietal cortices (Fig. 1; Huang et al., 2019).

Each participant completed three separate visits at the same time of day but separated by at least 1 week (M = 13.2 days, SD = 7.3 days; Fig. 1). Stimulation conditions were pseudorandomized and included two anodal (left parietal active, right parietal active) and one sham (right or left parietal, counterbalanced across participants) HD-tDCS sessions. Participants, as well as the researchers who analyzed the data, were kept blind as to the active/sham identity of the visit (i.e., double-blind design). Further, participants completed questionnaires to assess blinding and any side effects (e.g., itching after stimulation). These questionnaires suggested that only 15% of participants were able to correctly identify the active/sham conditions across all three sessions, which is just slightly higher than would be predicted by random chance (13%). During each active visit, participants underwent 20 minutes of 2.0 mA direct-current stimulation, plus a 30 second ramp-up period. No ramping-down period was used. During the sham visit, no actual stimulation was applied outside of the ramping-up period. To keep them cognitively engaged, participants performed a continuous performance test (CPT) during stimulation (Riccio et al., 2002). Following active/sham stimulation, participants were moved to the MEG recording chamber. By design, there was an interval of about 62–66 minutes from the end of the stimulation to the initiation of the MEG experiment (Fig. 1), which accords with the timeline considered suitable to capture offline tDCS effects (Kuo et al., 2013).

### 2.3 MEG experimental protocol

Participants completed a modified Posner task (Fig. 1; Posner, 1980). During the task, participants were seated in a magnetically shielded room with their head positioned within the MEG helmet-shaped sensor array. They were instructed to fixate on a crosshair presented centrally for 600 ms (±250 ms). Following that, a green bar (the cue) was presented either to the left or right of the crosshair for 100 ms. The cue appeared on a given side 50% of all trials and could either be valid (presented on the same side as the upcoming target, 50% of all trials) or invalid (presented on the opposite side relative to the target). At 300 ms (200 ms after cue offset), a target was presented on either the left or the right side of the crosshair for 1,400 ms, and this comprised a box with an opening on either its top (50% of trials) or bottom. Participants were instructed to respond as to whether the opening was on the top (right middle finger) or the bottom (right index finger) of the box. Each target variant appeared an equal number of times, and each trial lasted 2,300 ms (±250 ms). A total of 200 trials were used (100 valid, 100 invalid), leading to a total run-time of approximately 11 minutes. Trials were pseudo-randomly organized so that no more than three of the same target response or target/cue laterality pairs occurred in succession.

### 2.4 MEG data acquisition

All recordings were conducted in a two-layer magnetically shielded VACOSHIELD room (Vacuumschmelze, Hanau, Germany). With an acquisition bandwidth of 0.1–330 Hz, neuromagnetic responses were sampled continuously at 1 kHz using a MEGIN Triux Neo MEG system (Helsinki, Finland) with 306 sensors, including 204 planar gradiometers and 102 magnetometers. During data acquisition, participants were monitored via real-time audio-visual feeds from inside the shielded room. Each MEG dataset was individually corrected for head motion and subjected to noise reduction using the signal space separation method with a temporal extension (Taulu & Simola, 2006).

### 2.5 Structural MRI processing and MEG coregistration

Prior to MEG measurement, five coils were attached to the participant’s head and localized, together with the three fiducial points and scalp surface, with a 3D digitizer (FASTRAK, Polhemus Navigator Sciences, Colchester, VT, USA). Once participants were positioned for MEG recording, an electric current with a unique frequency label (e.g., 322 Hz) was fed to each of the coils. This induced a measurable magnetic field and allowed each coil to be localized in reference to the sensors throughout the recording session. As coil locations were also known with respect to head coordinates, all MEG measurements could be transformed into a common coordinate system. With this coordinate system, each participant’s MEG data were coregistered with their high-resolution T1-weighted structural brain data prior to source space analysis using BESA MRI (Version 2.0). Structural MRI data were aligned parallel to the anterior and posterior commissures and transformed into standardized space. Following source analysis, each participant’s MEG functional images were also transformed into standardized space and spatially resampled.

### 2.6 MEG pre-processing, time–frequency transformation, and sensor-level statistics

Eye blinks and cardio-artifacts were removed from the data using signal space projection (SSP), which was accounted for during source reconstruction (Uusitalo & Ilmoniemi, 1997). The continuous magnetic time series was divided into epochs (duration: 2,300 ms), beginning 600 ms prior to the onset of the stimuli and extending 1,700 ms afterward (i.e., -600 to 1,700 ms). The baseline period was defined as −600 to 0 ms. Epochs containing artifacts were removed based on a fixed threshold method, supplemented with visual inspection. Briefly, the amplitude and gradient distributions across all trials were determined per participant, and those trials containing the highest amplitude and/or gradient values relative to this distribution were rejected based on participant-specific thresholds. This approach was employed to minimize the impact of individual differences in sensor proximity and head size, which strongly affect MEG signal amplitude. Across all conditions and participants, the average amplitude threshold was 1,175.00 fT/cm (SD = 447.10), and the average gradient threshold was 200.49 fT/(cm*ms) (SD = 99.23). On average, 90.02 valid and 89.32 invalid trials per participant were used for further analysis, and the number of trials per participant did not significantly differ by task condition (*F*_1,31_ = 2.319, *p* = .138), stimulation montage (*F*_2,62_ = 2.290, *p* = .110), or their interaction (*F*_2,62_ = .867, *p* = .425) based on a 2 x 3 ANOVA. The percentage of included trials per stimulation condition was as follows: left active, 180.41 (90.21%); right active, 177.32 (88.66%); and sham, 180.29 (90.15%).

Epochs remaining after artifact rejection were transformed into the time–frequency domain using complex demodulation (Kovach & Gander, 2016), and the resulting spectral power estimations per sensor were averaged over trials to generate time–frequency plots of mean spectral density. The time–frequency analysis was performed with a frequency step of 2 Hz and a time step of 25 ms between 4 and 50 Hz to examine higher frequency activity (e.g., beta), as well as 1 Hz by 50 ms resolution from 2–20 Hz to better quantify lower frequency activity. These sensor-level data were normalized using the respective bin’s baseline power, which was calculated as the mean power during the −600 to 0 ms time period. The specific time–frequency windows used for source reconstruction were determined by statistical analysis of the sensor-level spectrograms across all participants and stimulation conditions using the entire array of 204 gradiometers. Specifically, paired-sample t-tests against baseline were initially conducted on each pixel, and the output spectrogram of t-values was thresholded at *p* < .05 to define time–frequency bins containing potentially significant oscillatory deviations across all participants and stimulation conditions. Time–frequency bins that survived the threshold were then clustered with temporally and/or spectrally neighboring bins that were also above the threshold (*p* < .05), and a cluster value was derived by summing the t-values of all data points in the cluster. Non-parametric permutation testing (1,000 permutations) was then used to derive a distribution of cluster values, and the significance level of the observed clusters was tested directly using this distribution (Ernst, 2004; Maris & Oostenveld, 2007).

### 2.7 MEG beamformer imaging and statistics

Cortical neural responses were imaged using the dynamic imaging of coherent sources (DICS) beamformer (Gross et al., 2001; Van Veen et al., 1997), which employs spatial filters in the time–frequency domain to calculate source power for the entire brain volume. Such images are typically referred to as pseudo-t maps, with units (pseudo-t) that reflect noise-normalized power differences (i.e., active vs. passive) per voxel. MEG pre-processing and imaging used the Brain Electrical Source Analysis (BESA; Version 7.1) software. Normalized source power was computed for the selected time–frequency windows (see Results section) over the entire brain volume per participant at 4.0 mm × 4.0 mm × 4.0 mm resolution. The resulting beamformer images were grand-averaged across all participants and HD-tDCS configurations (i.e., left active, right active, and sham) as a quality check and to determine the brain regions contributing most strongly to the oscillatory responses identified at the MEG sensor level. For further description of our analysis pipeline, see Wiesman and Wilson (2020).

Behavioral performance was assessed using two 2 x 3 repeated measures ANOVAs to identify the main effects of task condition (valid and invalid) and stimulation montage (left active, right active, and sham), as well as their interaction on accuracy and reaction time. For each participant, whole-brain functional maps were computed per task condition (valid and invalid) and stimulation montage (left active, right active, and sham) for the time–frequency windows that were significant in the sensor-level analyses. Since we were primarily interested in whether left versus right active parietal stimulation altered brain dynamics serving attention reorientation, we first subtracted the sham condition maps from each of the active conditions (i.e., left active sham and right active sham) per time–frequency window. These functional maps were then examined using repeated measures 2 x 2 ANOVAs to identify the main effects of task condition and stimulation montage, as well as their interaction. The resulting maps were thresholded at *p* < .005 and adjusted for multiple comparisons using a spatial extent threshold (i.e., cluster restriction; *k* > 10 contiguous voxels or >700 mm^3^; Poline et al., 1995; Worsley et al., 1996, 1999). Of note, follow-up analyses lowering the extent threshold indicated identical or very similar results down to five voxels. To probe the directionality of the significant effects, response amplitude values (pseudo-t) were extracted from the peak voxel of significant clusters and subjected to post hoc paired-samples t-tests. In addition, we conducted supplementary 2 x 3 repeated measures ANOVAs that included the sham condition, and these are included in the Supplementary Materials (see Figs. S1–S3). All statistical analyses were performed using *R* (R Core Team, 2020). Any values ±3 SD from the mean was considered an outlier and removed prior to statistical analysis, although follow-up analyses indicated that all significant effects in the whole-brain maps remained regardless of whether outliers were removed.

## 3 Results

All participants successfully completed the full study (i.e., all three visits), but five were excluded due to low trial counts in at least one of their visits. The remaining 34 participants (19 females) were 19–33 years old, with a mean age of 26.0 years (SD = 3.66 years).

### 3.1 Behavioral results

**Fig. 2.**
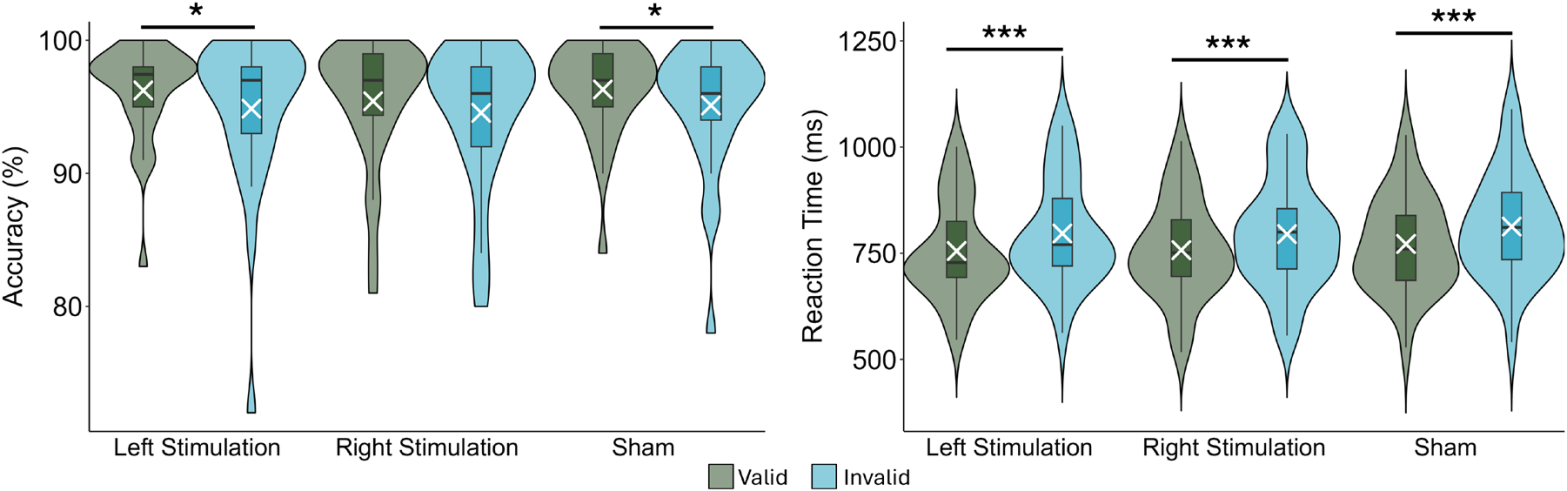
Behavioral performance on the Posner task. Stimulation montage is denoted on the x-axes, with accuracy (left) and reaction time (right) on the y-axes. Participants were significantly more accurate on valid trials than on invalid trials following left stimulation and sham. Participants were significantly faster to respond during valid trials than during invalid trials across all stimulation montages. Box plots reflect quartiles, with the white X’s showing the mean and the violins reflecting the probability density. Error bars reflect the SEM. *p < .05, ***p < .001.

To investigate the effect of task condition and stimulation montage on behavioral performance (i.e., accuracy and reaction time), we used two 2 x 3 repeated measures ANOVAs (Fig. 2). There were significant main effects of task condition for both accuracy (*F*_1,28_ = 9.131, *p* = .005) and reaction time (*F*_1,28_ = 127.97, *p* < .001), such that accuracy was higher and reaction times were shorter during valid trials than invalid trials. The average accuracy and reaction time validity effects (invalid–valid; Vossel et al., 2006) were -1.12% (SD = 1.39%) and 39.52 ms (SD = 8.83 ms). In contrast, there were no main effects of stimulation montage or task condition by stimulation montage interactions for accuracy or reaction time.

### 3.2 MEG sensor-level analysis

**Fig. 3.**
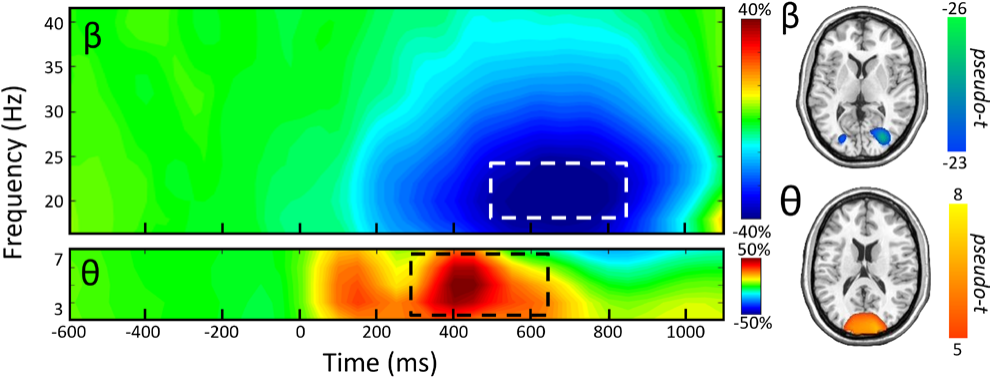
MEG sensor-level spectrograms showing oscillatory responses during the Posner task. (Left): Grand-averaged time–frequency spectrograms of MEG sensors exhibiting one or more significant oscillatory responses, with beta on top and theta on the bottom. In each spectrogram, frequency (Hz) is shown on the y-axis and time (ms) is on the x-axis. All signal power data are expressed as percentage difference from baseline, with color legends shown to the right of the spectrograms. Dashed boxes indicate the time–frequency windows that significantly differed from baseline and were subjected to beamforming. (Right): Grand-averaged beamformer images (pseudo-t) across all participants, conditions, and HD-tDCS montages per oscillatory response. Separate color scale bars are shown for each.

While strong theta and beta responses were observed after cue onset, the goals of this study were to examine oscillations associated with the attentional reorienting process. Thus, our statistical analyses focused on neural activity during the target period (i.e., starting 300 ms after cue onset). These analyses revealed two spectrally specific oscillatory responses in MEG gradiometers near the parietal and occipital cortices across all participants, conditions, and stimulation montages. Statistical testing of the time–frequency spectrograms showed robust decreases in beta power (18–24 Hz, *p* < .001, corrected) from about 500–850 ms. In addition, theta (3–7 Hz, *p* < .001, corrected) oscillations were stronger from about 300–650 ms (Fig. 3).

### 3.3 Functional mapping analysis

**Fig. 4.**
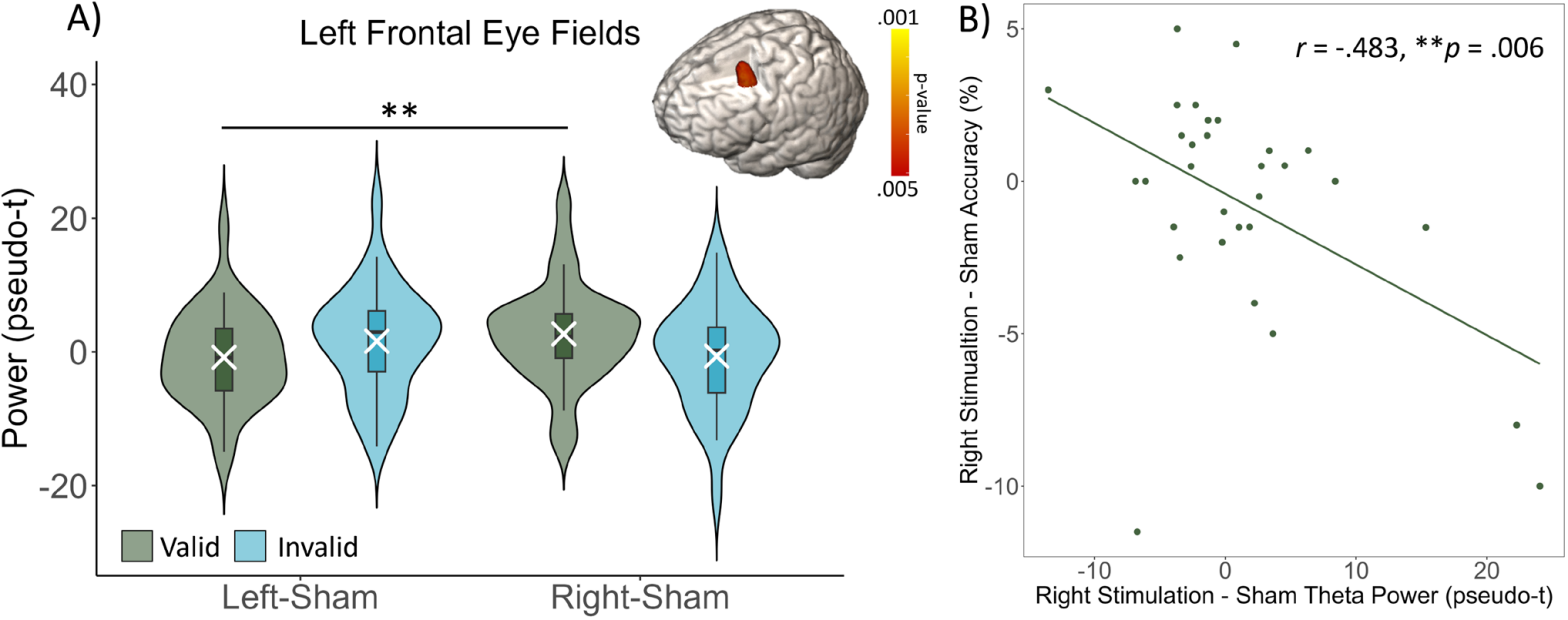
Interaction between stimulation montage and task condition on theta oscillations. (A) Theta power was significantly increased following right compared with left stimulation during valid trials in the superior frontal sulcus near the left frontal eye fields. Box plots reflect quartiles, with the white X’s showing the mean and the violins reflecting the probability density. Error bars reflect the SEM. (B) Following right stimulation, theta power was negatively correlated with accuracy. **p < .01.

**Fig. 5.**
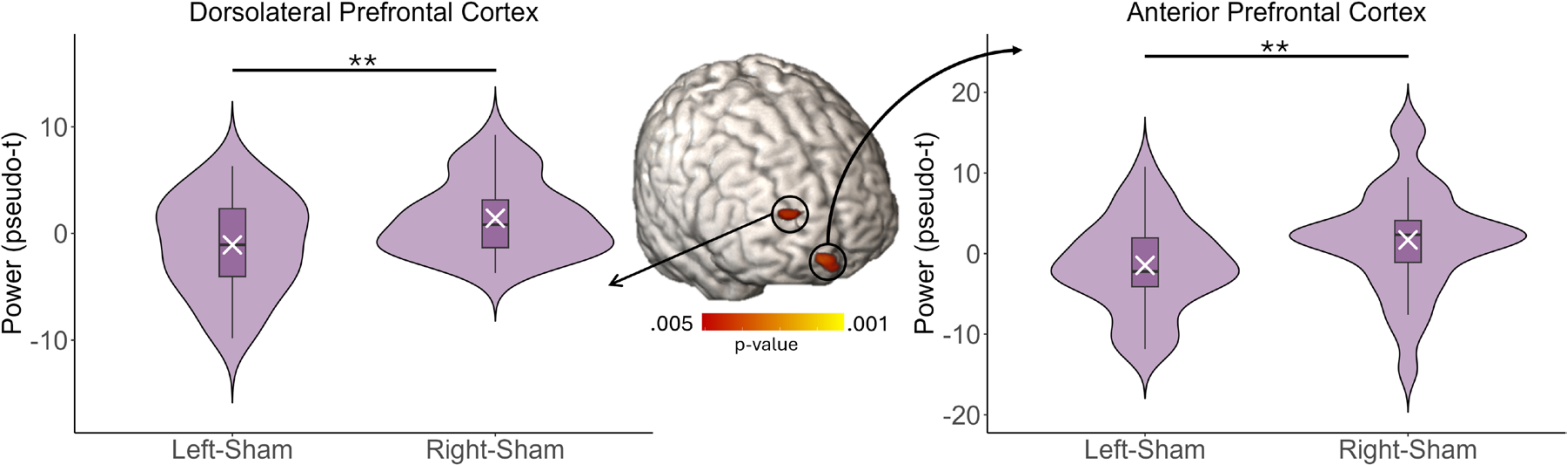
Main effects of stimulation montage on theta oscillations. Theta power was significantly stronger following right compared with left stimulation in the dorsolateral prefrontal cortex and the anterior prefrontal cortex. Box plots reflect quartiles, with the white X’s showing the mean values and the violins showing the probability density function. Error bars reflect the SEM. **p < .01.

To identify the spatial origins of these sensor-level oscillatory responses, the previously mentioned time–frequency windows of interest were imaged using a beamformer per task condition and stimulation montage. We then subtracted the sham condition images from each active condition images per time–frequency window of interest (see Methods section). These maps were then probed using whole-brain 2 x 2 repeated measures ANOVAs per oscillatory response, with post hoc paired-samples t-tests to determine the directionality of interactions. Regarding theta oscillations, there was a significant stimulation by task condition interaction effect in the superior frontal sulcus near the left frontal eye fields (*F*_1,31_ = 14.560, *p* < .001; Fig. 4). This interaction indicated that during valid trials, the theta response was significantly stronger following right HD-tDCS than following left (*t*(31) = -3.421, *p* = .002). In addition, there was a significant main effect of task condition in the midcingulate region (*F*_1,32_ = 14.987, *p* < .001), with participants exhibiting significantly stronger responses during valid than during invalid trials. There were also significant main effects of stimulation montage in the dorsolateral prefrontal cortex (*F*_1,31_ = 9.193, *p* = .005) and in the anterior prefrontal cortex (*F*_1,30_ = 12.454, *p* = .001), such that theta oscillations were significantly stronger following right than following left stimulation (Fig. 5). Lastly, to determine whether this neural activity was linked with behavioral metrics, a Pearson’s correlation was conducted between theta power at the peak voxel in the interaction cluster and mean accuracy across both task conditions, separately for each stimulation condition, with one outlier exclusion for accuracy. Following right stimulation, this analysis revealed a significant negative association between accuracy and theta power in the left frontal eye fields (*r* = -.483, *p* = .006), such that as theta power increased, accuracy decreased. There was no correlation between left stimulation and accuracy.

**Fig. 6.**
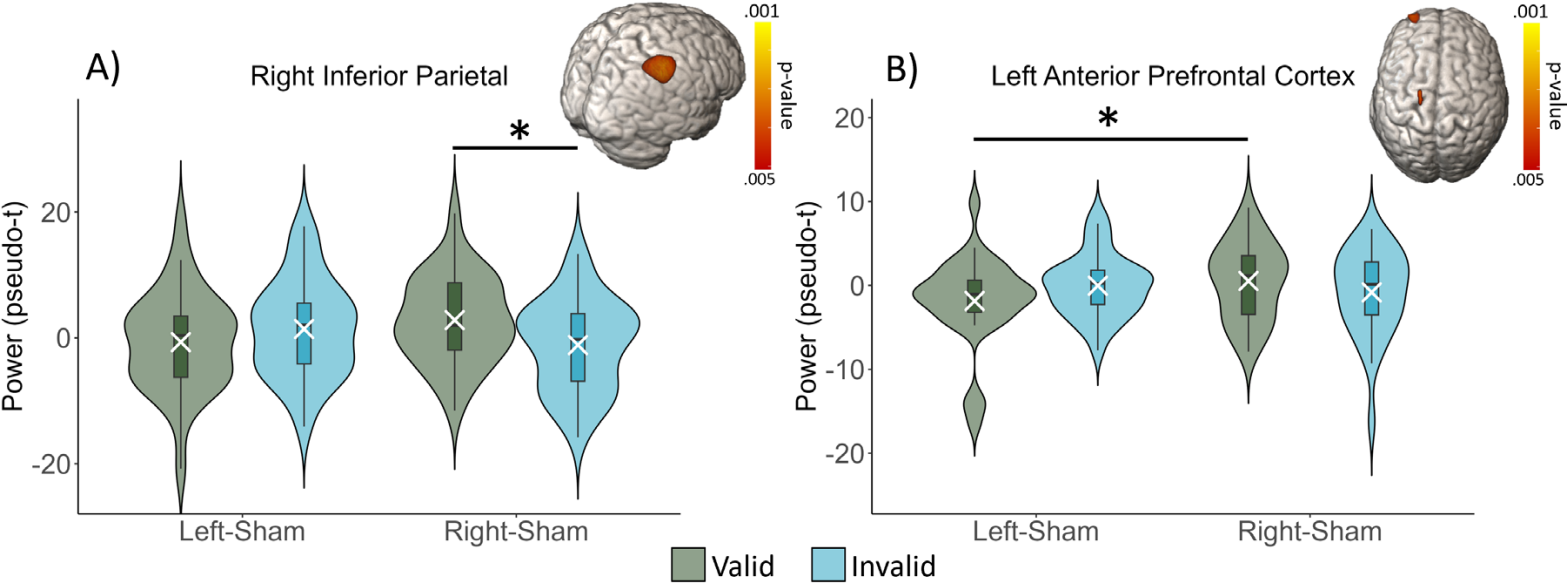
Modulation of beta oscillations by stimulation montage and task condition. (A) In the inferior parietal cortex, beta oscillations were stronger (i.e., more negative relative to baseline) during invalid than during valid trials following right stimulation. (B) In the left anterior prefrontal cortex, beta oscillations were stronger during valid trials following left than following right stimulation. Box plots reflect quartiles, with the white X’s showing the mean values and the violins reflecting the probability density function. Error bars reflect the SEM. *p < .05.

In the beta band, there were significant stimulation montage by task condition interaction effects in the right inferior parietal cortex (*F*_1,31_ = 19.133, *p* < .001; Fig. 6), left anterior prefrontal cortices (*F*_1,29_ = 9.466, *p* = .005), and the left primary motor cortices (*F*_1,30_ = 9.716, *p* = .004). The primary motor peak was not explored further because the focus of this study was on cognitive effects, not movement effects. In the inferior parietal cortex, there was a stronger beta response (i.e., more negative) during invalid than during valid trials (*t*(31) = -2.586, *p* = .015) following right stimulation. In contrast, in the left anterior prefrontal cortex, there were significantly stronger beta responses during valid trials following left than following right stimulation (*t*(29) = -2.379, *p* = .024). There were also two main effects of task condition, with peaks in the primary motor region (*F*_1,30_ = 12.084, *p* = .002) and the temporoparietal junction (TPJ; *F*_1,30_ = 11.504, *p* = .002), and reflecting stronger beta responses during valid than during invalid trials. There were no main effects of stimulation montage.

## 4 Discussion

The purpose of this study was to identify the effects of left versus right inferior parietal HD-tDCS on the neural oscillations serving attentional reorientation in healthy adults. We hypothesized that right parietal stimulation would have a stronger effect on the neural processes serving attention reorientation, and this was partially supported by our results. Regarding the neural data, significant task condition by stimulation montage effects in the theta band were found in the left frontal eye fields, such that theta power during valid trials was significantly stronger following right than following left stimulation. In the beta range, there were significant interaction effects in the inferior parietal cortex and the anterior prefrontal cortex. Post hoc testing indicated that in the inferior parietal cortex, beta power was significantly stronger (i.e., more negative) during invalid trials than during valid trials following right stimulation, while in the anterior prefrontal cortex, beta power during valid trials was significantly stronger following left than following right stimulation. Lastly, there were significant main effects of task condition across multiple brain regions in the theta and beta bands, as well as main effects of stimulation montage on theta oscillations in the dorsolateral prefrontal and anterior prefrontal cortices. Below, we discuss the implications of these findings for understanding the impact of HD-tDCS on neural function and the differential roles of left versus right parietal cortices in attention reorientation.

One of our most important findings was the significant interaction effect on theta oscillations in the left frontal eye fields. This region has been implicated in attention reorientation in many previous studies, as increases in frontal theta activity have been found during invalid compared with during valid trials, and activity due to reorienting has been shown in an overlapping area of the left middle frontal gyrus (Arif, Spooner, Wiesman, Embury, et al., 2020; Spooner et al., 2020; Vossel et al., 2006). Our results showed that right inferior parietal stimulation was associated with stronger theta responses during valid trials than left stimulation. Given the unique effect on valid trials, it could be that this processing reflects more assessment level operations preceding orientation (and not reorientation). Further, we found that there was a correlation between theta power and accuracy following right stimulation but not following left stimulation. Our results may indicate that right inferior parietal stimulation alters normal processing in the left frontal eye fields during attention reorientation, while left inferior parietal stimulation has a less profound effect. Of note, the right inferior parietal cortex is known to play a much larger role in attention reorientation (Igelström & Graziano, 2017; Jing et al., 2023; Numssen et al., 2021), so this region could be more susceptible to modulation than the left inferior parietal cortex, and thus be associated with larger changes in other brain regions contributing to attentional orienting, such as the left superior frontal sulcus and frontal eye fields. Interestingly, we also observed main effects of stimulation in areas of the right dorsolateral prefrontal and anterior prefrontal cortices, which indicated that across both valid and invalid conditions, theta oscillations were stronger in these areas following right inferior parietal relative to left stimulation. This further supports the notion that right inferior parietal stimulation may have a greater effect than left inferior parietal stimulation on other brain regions known to be critical during attentional orienting and reorienting.

We also observed significant task condition by stimulation montage interaction effects on beta oscillations in the right inferior parietal cortex and the left anterior prefrontal cortices. In the right inferior parietal cortex, beta responses were significantly stronger during invalid trials than during valid trials following right stimulation. As noted in the introduction, the inferior parietal cortex is widely known to be critical for attentional reorienting, and this was the motivation for stimulating these regions in the current study. Several studies have suggested that the inferior parietal cortices are especially important during the alerting phase (Fan et al., 2005, 2007; Périn et al., 2010), and the time course of the current responses would support this. Further, the beta interaction in the right inferior parietal cortex was driven by stronger responses during the invalid trials following right stimulation. Thus, this may indicate that right inferior parietal stimulation alters normal processing locally during attentional orienting or alerting, while left parietal stimulation does not, but further work is needed to isolate the precise origin. We additionally found a significant stimulation montage by task condition interaction in the ventral part of the anterior prefrontal cortex. The ventral frontal region has been tied to attentional reorientation through the ventral attention network (VAN), which is utilized in the detection of behaviorally relevant stimuli and the processing of invalid targets (Corbetta et al., 2008; Proskovec et al., 2018).

There were also many significant main effects of task condition throughout the theta and beta bands, in regions such as the TPJ, primary motor, anterior prefrontal, and other regions. The Posner task has been well studied in the past, and our findings relating to task condition were consistent with the literature (Arif, Wiesman, et al., 2020; Doricchi et al., 2010; Eckstein et al., 2002; Flores et al., 2009; Proskovec et al., 2018; Spooner et al., 2020; Springer et al., 2023; Vergallito et al., 2023). Thus, while tDCS can have effects on neural oscillations, the underlying processes serving attentional reorientation remain consistent.

Before closing, it is important to address some limitations of this study. First, stimulation studies in general are often limited as it is difficult to fully blind participants to the stimulation montage due to the physical sensation. However, our participants were unable to reliably distinguish between active and sham visits in their questionnaire data. Thus, we believe our approach in this study resulted in adequate blinding. Second, we did not observe behavioral differences by stimulation montage. Many previous tDCS studies have shown behavioral differences (Arif, Spooner, Wiesman, Proskovec, et al., 2020; Arif et al., 2021; McDermott et al., 2019; Wiesman et al., 2018), though many others have not (Arif et al., 2022, 2024; P. Huang et al., 2025; Son et al., 2025; Wilson et al., 2018), and there are likely a large number of contributing factors. One possibility is that the neural differences observed in the current study reflect compensatory activity that was necessary to maintain adequate task performance following the stimulation. Future work is clearly needed to identify the impact of the neural differences we observed following stimulation. Third, there was a delay of 62–66 minutes between the offset of stimulation and the onset of the Posner task during MEG. Although Kuo et al. (2013) and others have suggested effects lasting out to 2 hours or more, it is possible that the effects on neural oscillatory dynamics and behavior in the current study would have been stronger had the delay period been shorter (e.g., ~30 minutes). Thus, future work should utilize shorter and longer delays to determine the ideal time window for observing effects on the neural dynamics and behavior. In sum, the current study utilized MEG and offline HD-tDCS to show that left and right stimulation of the inferior parietal cortices modulate multispectral neural oscillations within brain regions that are critical for attentional reorientation. Our findings highlight the impact of lateralized parietal stimulation and the impact of this on the region’s critical role in attention reorientation. Future studies should further investigate the effects of parietal stimulation in conjunction with stimulation of other regions known to be essential to attentional reorientation with the goal of enhancing behavioral performance.

## Data and Code Availability

The data used in this article will be made publicly available through the COINS framework at the completion of the study (https://coins.trendscenter.org/).

## Supplementary Material

Supplementary Material
