# Supplementary material for: High-definition transcranial direct current stimulation of the right inferior parietal modulates oscillatory activity in higher-order regions serving attention reorientation
Source: Imaging Neurosci (Camb). 2025 Dec 9;3:IMAG.a.1054. doi: 10.1162/IMAG.a.1054 (PMC12690293; doi:10.1162/IMAG.a.1054)
Supplement: Supplementary Material [file IMAG.a.1054_supp.pdf]

Supplemental Materials:

Title: High-definition transcranial direct current stimulation of the right inferior parietal modulates oscillatory activity in higher-order regions serving attention reorientation

Tara D. Erker, Yasra Arif, Jason A. John, Kellen M. McDonald, Hannah J. Okelberry, Kennedy A. Kress, Giorgia Picci, Tony W. Wilson

Supplementary Results

The main focus of this investigation was on how the laterality of parietal stimulation affects the underlying whole-brain oscillatory dynamics. Thus, to decrease complexity and limit the number of statistical comparisons in the primary analyses, we subtracted the sham data from each active stimulation condition prior to statistical analyses. However, for completeness, we also present analyses below with the sham data in the same model. To this end, 2 x 3 repeated-measures ANOVAs were conducted, with task condition as a two-level within-subjects factor (valid and invalid) and stimulation montage as a three-level within-subjects factor. These results and figures for significant findings are provided below.

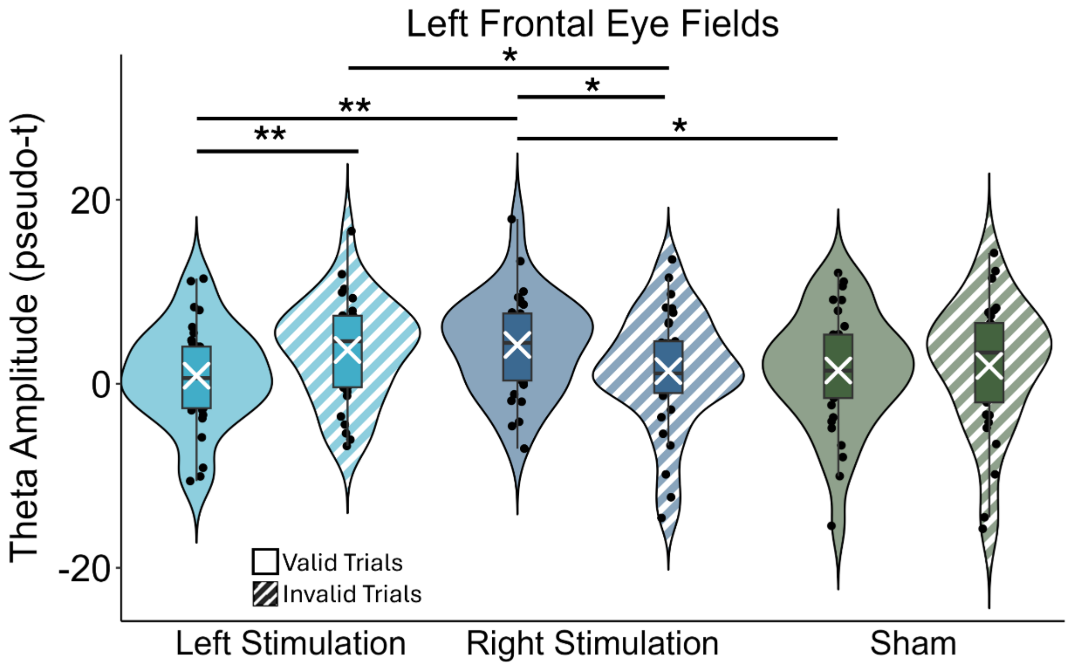

Supplementary Figure 1 - Theta activity is modulated by stimulation montage and task condition. The 2 x 3 repeated measures ANOVA indicated a task condition by stimulation montage interaction ($F = 5.868$, $p = .005$) on theta oscillations in the left frontal eye fields. Neither the task condition nor the stimulation montage main effects were significant. Post-hoc testing indicated that during valid trials, there were

significantly stronger theta responses following right stimulation compared to left stimulation and sham. During invalid trials, there were significantly stronger theta oscillations following left stimulation compared to right stimulation. In addition, significantly stronger theta responses were observed during invalid compared to valid trials following left stimulation, while the inverse was observed following right stimulation, with significantly stronger theta responses during valid compared to invalid trials. Box plots reflect quartiles, with the white X's showing the mean and the violins reflecting the probability density. Error bars reflect the SEM. * $p < .05$, ** $p < .01$

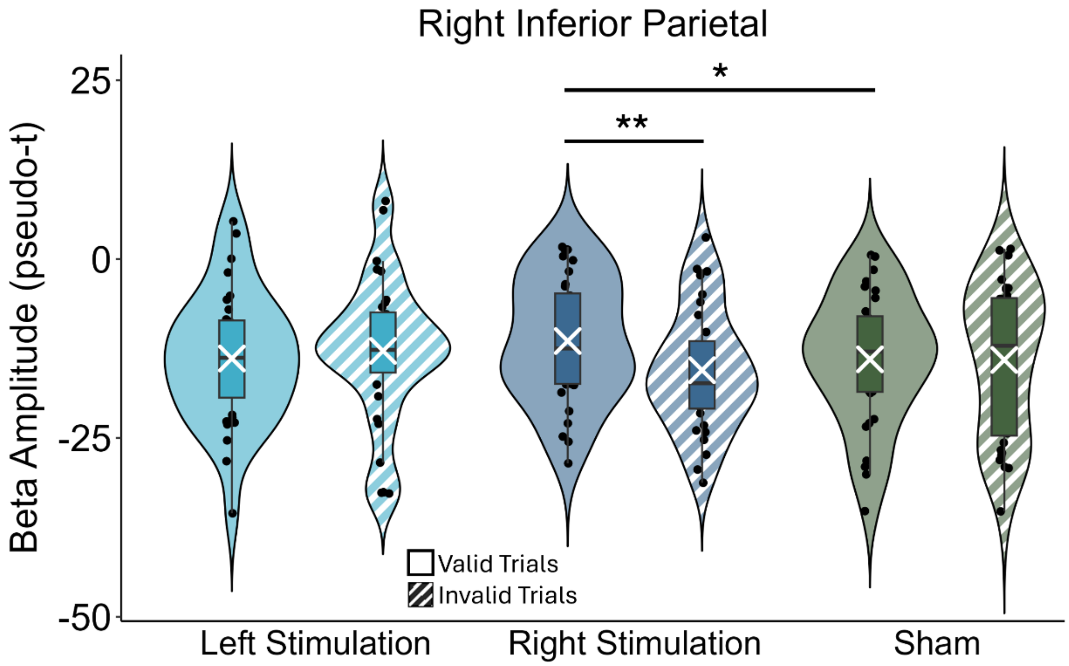

Supplementary Figure 2 – Interaction between stimulation montage and task condition on beta amplitude in the right inferior parietal cortex. The 2 x 3 repeated measures ANOVA indicated a task condition by stimulation montage interaction ($F = 7.440$, $p = .001$) on beta responses in the right inferior parietal. Neither the task condition nor the stimulation montage main effects were significant. Post-hoc testing indicated that following right stimulation beta oscillations were significantly stronger (i.e., more negative) during invalid compared to valid trials in the right inferior parietal cortex. In addition, during valid trials, beta responses were significantly weaker following right stimulation compared to sham. Box plots reflect quartiles, with the white X's showing the mean and the violins reflecting the probability density. Error bars reflect the SEM. * $p < .05$, ** $p < .01$

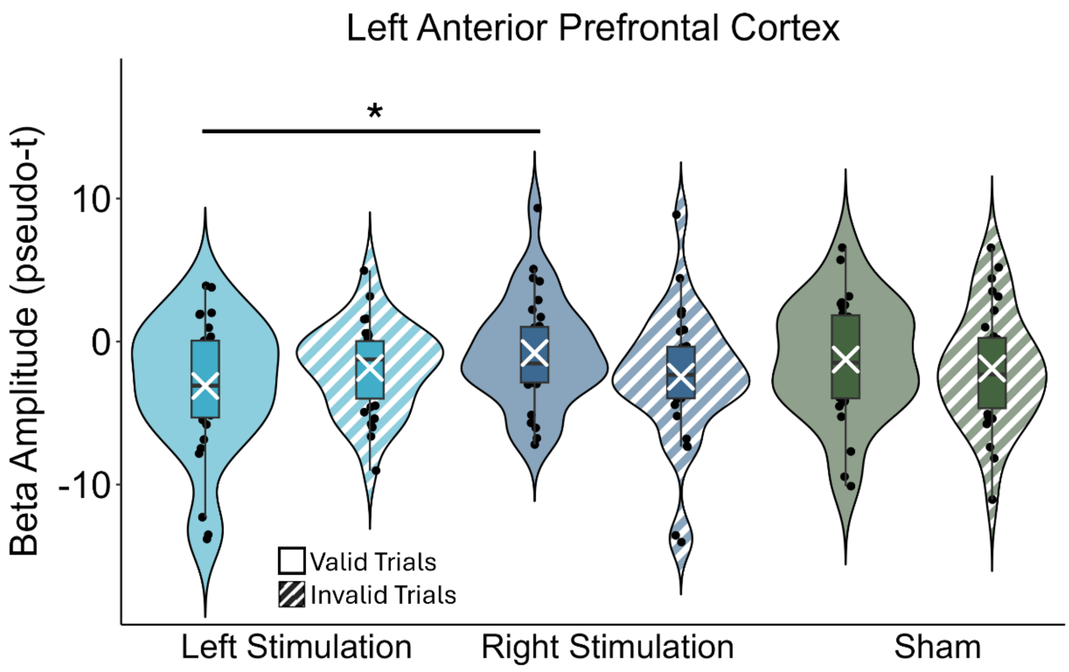

Supplementary Figure 3 – Effects of stimulation montage and task condition on beta oscillations in the left anterior prefrontal cortex. The 2 x 3 repeated measures ANOVA indicated a task condition by stimulation montage interaction ($F = 4.014$, $p = .023$) in the left anterior prefrontal cortex. Neither the task condition nor the stimulation montage main effects were significant. Post-hoc testing showed that during valid trials, beta oscillations were significantly stronger following left compared to right stimulation. Box plots reflect quartiles, with the white X's showing the mean and the violins reflecting the probability density. Error bars reflect the SEM. * $p < .05$
